# Consumption and cost trends of EGFR TKIs: influences of reimbursement and national price negotiation

**DOI:** 10.1186/s12913-022-07868-9

**Published:** 2022-04-01

**Authors:** Di Wu, Jianxiang Xie, Huizhen Dai, Wentong Fang

**Affiliations:** 1 Department of Pharmacy, The First Affiliated Hospital of Nanjing Medical University, No 300 Guangzhou Road, Nanjing City, Jiangsu Province 210029 People’s Republic of China; 2 Department of Pharmacy, Jiangsu Medicine Information Institute, Nanjing, 210029 China

**Keywords:** Tyrosine kinase inhibitors, Consumption, Drug reimbursement, National negotiation, Generic drug

## Abstract

**Background:**

Epidermal growth factor receptor (EGFR) tyrosine kinase inhibitors (TKIs) have been widely used in the treatment of EGFR mutation non-small-cell lung cancer. The Chinese government has made great efforts to improve the availability and affordability of these drugs. The aim of this study was to investigate the trends in the consumption and cost of EGFR TKIs in Nanjing, a developed city in China, and evaluate the influence of health insurance coverage and national price negotiation on drug consumption.

**Methods:**

Data about EGFR TKIs applications in 2010–2019 were extracted from Jiangsu Medicine Information Institute. Five types of EGFR TKIs were included. Consumption was expressed in defined daily doses (DDDs) and expenditure. The correlation between defined daily cost (DDC) and DDDs was analyzed by Pearson's correlation test.

**Results:**

The DDC, number of DDDs and expenditure of EGFR TKIs changed little from 2010 to 2015. National price negotiation was initiated as a policy and low-price generic gefitinib came into the market in 2016. Three types of EGFR TKIs moved into the coverage of the national health insurance since 2017. Hence, the DDC decreased, and the number of DDDs increased significantly year by year since 2016. The first generation TKIs always made up of comprised the majority of the total consumption. The predominantly prescribed TKIs were gefitinib and icotinib. DDC was negatively correlated with the number of DDDs. The number of DDDs increased significantly after health insurance enrollment, price negotiation and generic drug replacement.

**Conclusion:**

The consumption of EGFT TKIs has increased and the DDC of EGFR TKIs has decreased since 2016. These trends may be attributed to drug reimbursement, price negotiation and generic drug replacement. Further efforts are needed to translate the high consumption of EGFR TKIs into clinical benefits.

**Supplementary Information:**

The online version contains supplementary material available at 10.1186/s12913-022-07868-9.

## Background

According to the World Health Organization (WHO) Global Cancer Observatory (GLOBOCAN) [1], lung cancer brings with the highest age-standardized mortality (18.6 per 100,000), as evidenced by the 1.76 million deaths worldwide in 2018. Fortunately, the treatment of lung cancer has witnessed several major breakthroughs in the past decade. Targeted therapy and immunotherapy have become the first line options for selected patients. Epidermal growth factor receptor (EGFR) tyrosine kinase inhibitors (TKIs) have been widely used for EGFR mutation lung cancer patients. Routine EGFR TKIs, including gefitinib, erlotinib, afatinib, and osimertinib, can shrink tumor, prolongate overall survival (OS) and improve quality of life compared to standard chemotherapy [2].

Activating EGFR mutations occur in 10–20% of patients with non-small-cell lung cancer (NSCLC) in North America and Europe and up to 60% in Asia [3]. Inequalities exist in the availability and affordability of these drugs. Carbonnaux M et al. [4] conducted a prospective survey to evaluate EGFR-TKI availability in 192 countries in 2014. Respectively, erlotinib, gefitinib, afatinib and icotinib were routinely available in 86%, 73%, 38%, and 5% of the responding 74 countries. In these countries, the cost exceeded 1000 dollars per month in 39%, 35%, and 50% of patients using erlotinib, gefitinib, and afatinib under public or private mandatory health insurance systerms.

It is necessary to improve the availability and affordability of EGFR TKIs considering the high incidence of EGFR mutation (about 60%) in Chinese NSCLC patients [3]. Before 2016, gefitinib, erlotinib, and icotinib were on the Chinese market but not covered by the national insurance systerm (Table 1). The Chinese government held the first round of national price negotiation with manufacturers in 2016.

**Table 1:** Information about the EGFR-TKIs

Drugs	Generation	Manufacturer	Launch Date	Reimbursement Date	Price negotiation
Gefitinib (original)	First	AstraZeneca (UK)	Feb 2005	Jan 2017	¥ 510.00 to ¥ 235.80 (Jul 2016)
					¥ 235.80 to ¥ 228.00 (Sep 2018)
Gefitinib (generic)	First	Qilu (China)	Feb 2017	Jan 2017	¥ 176.00 to ¥ 158.40 (Apr 2018)
					¥ 158.40 to ¥ 80.00 (Apr 2019)
					¥ 80.00 to ¥ 27.50 (Dec 2019)
Erlotinib	First	Roche (USA)	Mar 2007	Jul 2017	¥ 601.24 to ¥ 195.00 (Jul 2017)
					¥ 195.00 to ¥ 182.25 (Jan 2019)
					¥ 182.25 to ¥ 81.00 (Sep 2019)
Icotinib	First	Betta (China)	Aug 2011	Jan 2017	¥ 429.28 to ¥ 396.43 (Jan 2015)
					¥ 396.43 to ¥ 199.86 (July 2016)
					¥ 199.86 to ¥ 192.15 (Jan 2019)
Afatinib	Second	BoehringerIngelheim (German)	Feb 2017	Jan 2019	_______
Osimertinib	Third	BoehringerIngelheim (German)	Mar 2017	Jan 2019	_______

In 2017, three types of TKIs (gefitinib, icotinib, and erlotinib) were enrolled in the reimbursement list, with a reimbursement rate of 50% [5]. The generic gefitinib was marketized in December 2016, and its monthly cost was significantly lower than that of the original drugs (¥ 5280 VS ¥ 7074) [6]. Several rounds of national price negotiation have been held since 2017, and the monthly cost of TKIs has reduced gradually [5, 7, 8]. In January 2019, afatinib and osimertinib were covered by the national health insurance [5, 7] (Table 1).

In China, the coverage rate of the basic medical insurance has increased from 90% in 2010 to 95% in 2019 [9]. Therefore, the consumption and expenditure of EGFR TKIs might have undergone some changes. Nanjing, a city in eastern China, has a developed economy. The aim of this study was to investigate the trends in the consumption and cost of EGFR TKIs in Nanjing from 2010 to 2019, and to evaluate the influence of medical insurance and price negotiation on these trends.

## Methods

### Data sources

The application data of EGFR TKIs (gefitinib, erlotinib, icotinib, afatinib, and osimertinib) were provided by the Jiangsu Medicine Information Institute [10]. In China, EGFR TKIs, which belong to prescription drugs, are mainly sold by hospital pharmacies. If these drugs were covered by medical insurance, only drugs sold by hospital pharmacies can be reimbursed. Hence, the consumption of hospital pharmacies could present the total consumption of EGFR TKIs.

Five kinds of EGFR TKIs are used in Nanjing, and they are gefitinib, erlotinib, icotinib, afatinib, and osimertinib before 2020. Erlotinib, afatinib, and osimertinib are imported drugs. Icotinib is invented by a Chinese pharmaceutical company named BettaPharm in Zhejiang Province. Gefitinib was firstly invented and sold by AstraZeneca Pharmaceutical Co Ltd. Generic gefitinib launched on the market in 2016 after the expiration of the patent term. The details of EGFR TKIs used in Nanjing are listed in Table 1.

### Statistical analysis

The institute reported the consumption of TKIs in terms of grams and prices. We estimated them in terms of defined daily doses (DDDs) [11]. The defined daily dose (DDD) is a statistical unit defined by the WHO Collaborating Centre (WHOCC) for Drug Statistics Methodology [12]. The expenditure was recorded in Yuan (Ұ).

In our study, DDDs was calculated with the following formula:

- The number of DDDs = (∑(Total dose used in number of gram)/DDD)
- Expenditure = (retail price per package)*(consumption amount in number of package)
- Daily dose cost (DDC) was the cost of per DDD drugs.
- DDC = expenditure/(the number of DDDs)
- Daily cost = (DDD)*(DDC)

Correlation between DDDs and DDC was analyzed by applying Pearson's correlation test and linear regression analysis in SPSS version 21 (IBM Corporation, Armonk, New York, USA). *p* < 0.05 was considered to show a significant linear correlation. All figures were made by using Prism 8.0 (GraphPad Software).

## Results

### Consumption and cost trends of EGFR TKIs

**Fig. 1:**
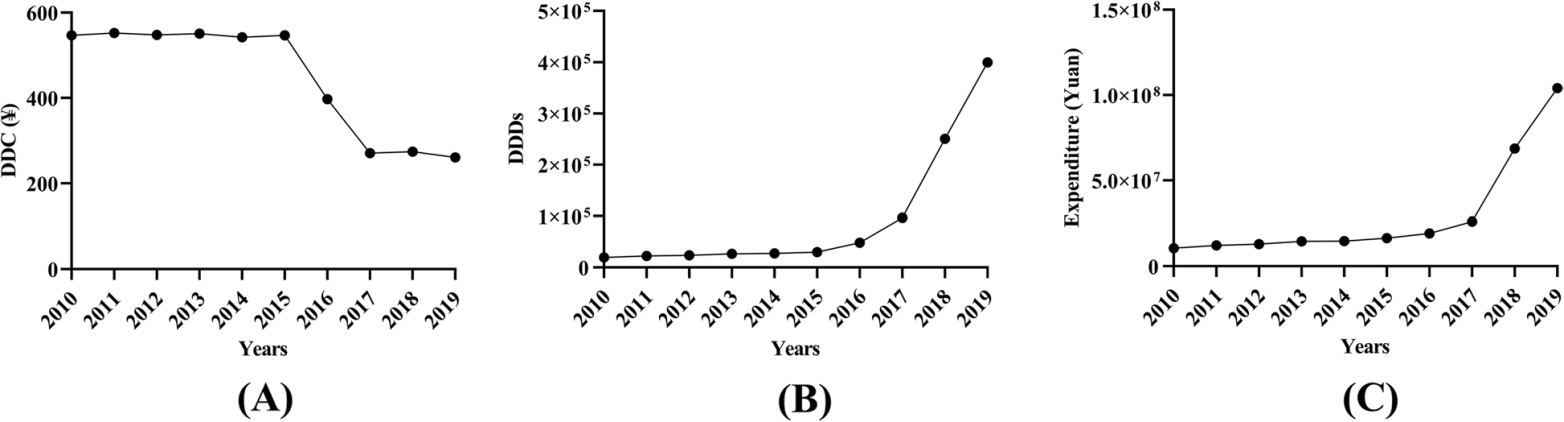
Consumption of EGFR TKIs in Nanjing from 2010 to 2019. **A** DDC of EGFR TKIs; (**B**) DDDs of EGFR TKIs; (**C**) Expenditure on EGFR TKIs. DDC, defined daily dose; DDDs, defined daily doses

The number of DDDs of all TKIs changed slightly from 2010 to 2015, but kept increasing since 2016. The number of DDDs increased by 69.0% in 2016, 100.0% in 2017, 159.4% in 2018, and 59.5% in 2019 (Fig. 1A). Accordingly, expenditure also kept increasing significantly (Fig. 1B). The average DDC of all TKIs changed slightly from 2010 to 2015, but decreased significantly from 2016 to 2017 (Fig. 1A). The DDC decreased by 27.3% in 2016 and 31.9% in 2017 (Fig. 1C). The average DDC changed little in 2018 and 2019 (Fig. 1C).

### Consumption of three generations of EGFR TKIs

**Fig. 2:**
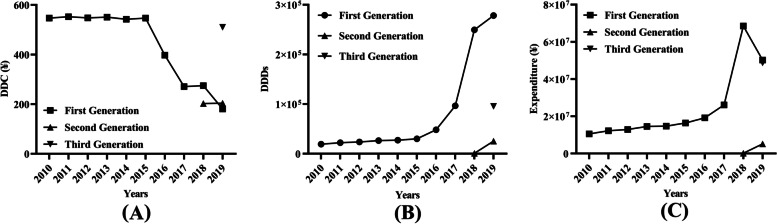
Consumption of three generation of EGFR TKIs in Nanjing from 2010 to 2019. **A** DDC of three generation of EGFR TKIs; (**B**) DDDs of three generation of EGFR TKIs; (**C**) Expenditure of three generation of EGFR TKIs. DDC, defined daily dose; DDDs, defined daily doses

The number of DDDs (Fig. 2A), expenditure (Fig. 2B) and DDC of the first-generation TKIs changed little from 2010 to 2015. The DDC of the first-generation TKIs decreased gradually (Fig. 2C), while their number of DDDs increased year by year since 2016 (Fig. 2A). Overall, the number of DDDs and expenditure of the first-generation TKIs made up always took most of the total consumption from 2010 to 2019. The second-generation TKI (afatinib) entered the market since 2018, and the number of DDDs (Fig. 2A) and expenditure (Fig. 2B) increased significantly in 2019. The third-generation TKI (osimertinib) was launched in 2019, and its DDC was significantly higher than those of other TKIs.

### Consumption of each type of EGFR TKIs

**Fig. 3:**
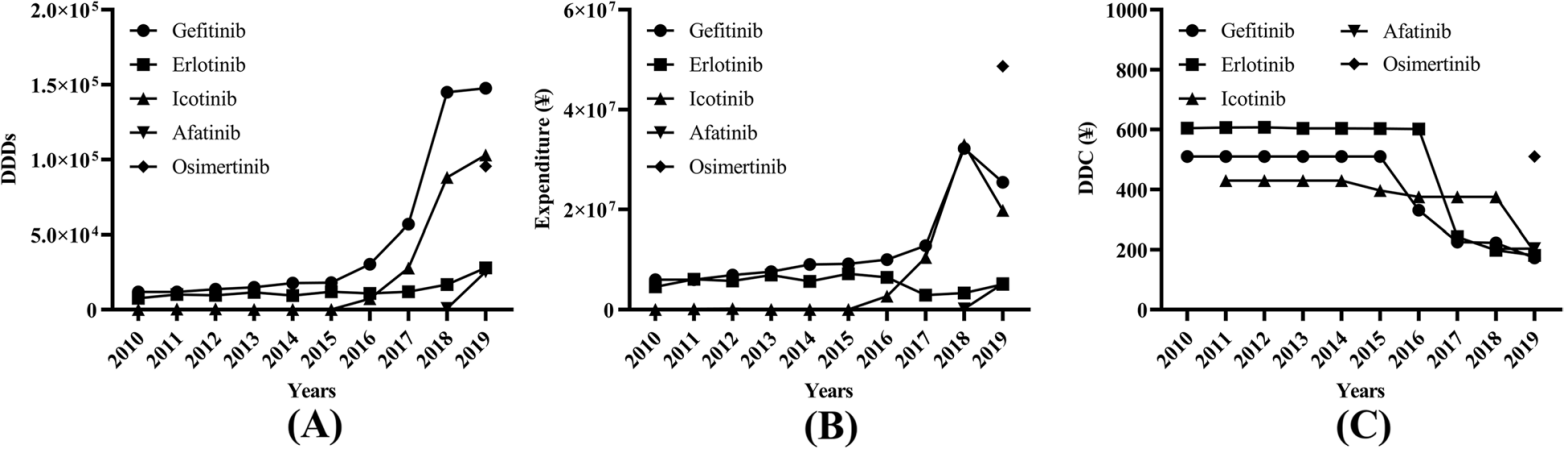
Consumption of five types of EGFR TKIs in Nanjing from 2010 to 2019. **A** DDC of five types of EGFR TKIs; (**B**) DDDs of five types of EGFR TKIs; (**C**) Expenditure of five types of EGFR TKIs. DDC, defined daily dose; DDDs, defined daily doses

Before 2018, gefitinib, erlotinib, and icotinib were on the market. Their number of DDDs, expenditure, and DDC changed slightly from 2010 to 2015. The number of DDDs of these three drugs showed an ascending trend (Fig. 3A), while the DDC showed a descending trend since 2016 (Fig. 3C). The number of DDDs of gefitinib, which was the most widely applied type, accounted for more than 50% of the total number of DDDs from 2010 to 2018 (Fig. 3A). In 2019, the number of DDDs of gefitinib slightly increased, but only made up 36.96% of the total number of DDDs (Fig. 3A). Icotinib came into use in 2011, and its consumption was low from 2011 to 2015. The number of DDDs increased significantly (Fig. 3A), but the DDC of icotinib decreased annually from 2016 to 2019 (Fig. 3C). The number of DDDs of erlotinib has been always less than that of gefitinib (Fig. 3A). The DDC of erlotinib decreased significantly in 2017(Fig. 3C), but its number of DDDs increased slightly (Fig. 3A). Afatinib came into market in 2018 and osimertinib in 2019, but their consumptions were relatively low (Fig. 3A and 3B). The DDC of osimertinib was the highest in all the TKIs (Fig. 3C).

### Relationship between DDC and DDDs

**Fig. 4:**
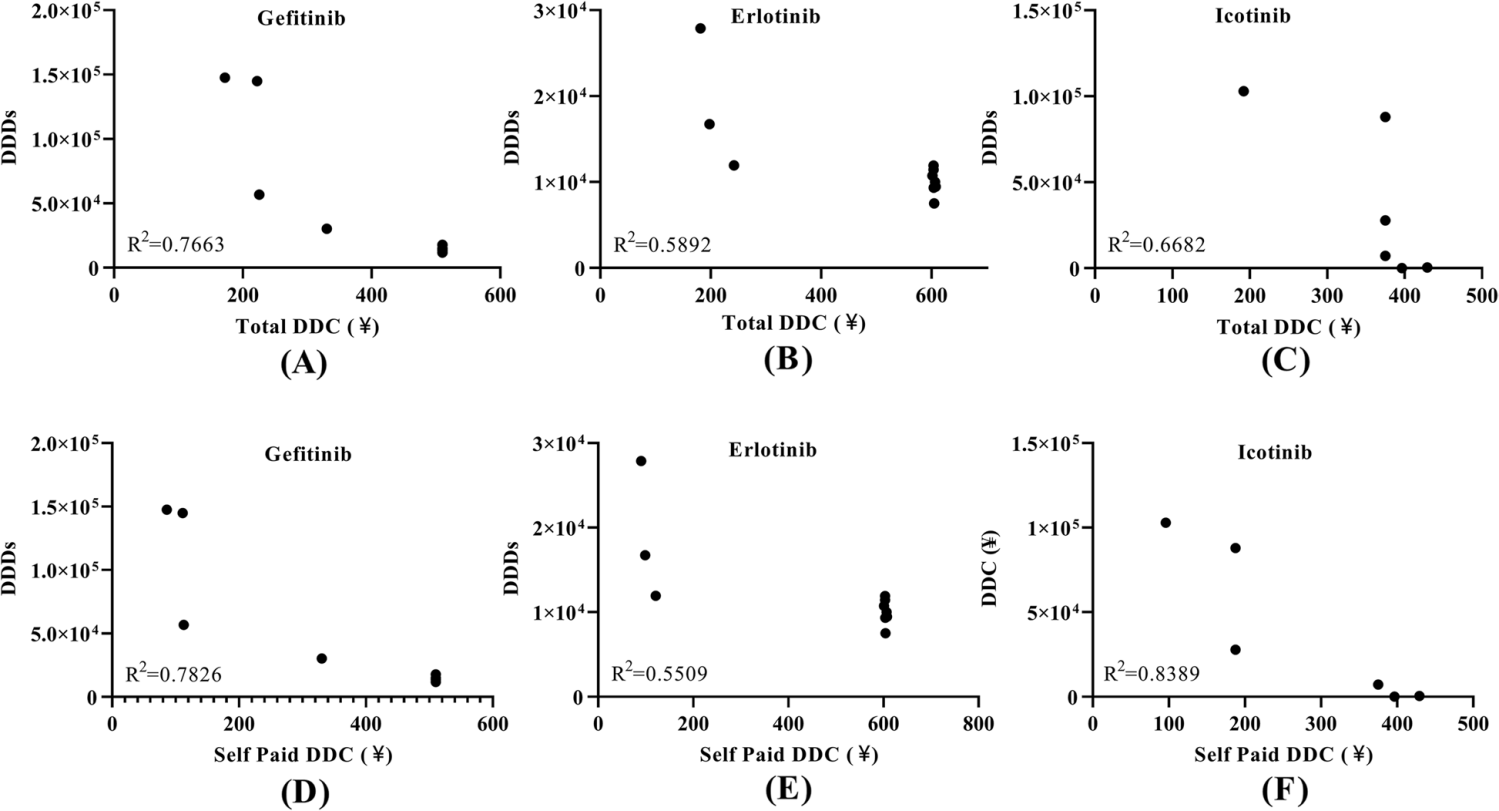
Correlation of DDC and DDDs of EGFR TKIs in Nanjing from 2010 to 2019. **A** Correlation between total DDC and DDDs of Gefitinib in Nanjing from 2010 to 2019; (**B**) Correlation between total DDC and DDDs of Erlotinib in Nanjing from 2010 to 2019; (**C**) Correlation between total DDC and DDDs of Icotinib in Nanjing from 2010 to 2019; (**D**) Correlation between self-paid DDC and DDDs of Gefitinib in Nanjing from 2010 to 2019; (**E**) Correlation between self-paid DDC and DDDs of Erlotinib in Nanjing from 2010 to 2019; **(F**) Correlation between self-paid DDC and DDDs of Icotinib in Nanjing from 2010 to 2019. DDC, defined daily dose; DDDs, defined daily doses

Over the past decade, the price of the first-generation TKIs has reduced, hence we analyzed the relationship between their DDC and number of DDDs. The DDC of gefitinib decreased gradually, while the number of DDDs increased continuously since 2016. The DDC of gefitinib had a negative correlation with its number of DDDs (*R*^2^ = 0.7663, *P* = 0.0009) (Fig. 4A). A negative correlation existed between the DDC and number of DDDs for erlotinib (*R*^2^ = 0.5892, *P* = 0.0095, Fig. 4B) and icotinib (*R*^2^ = 0.6682, *P* = 0.0071, Fig. 4C).

As gefitinib, icotinib and erlotinib were enrolled in the national insurance in 2017, self-paid cost (out of pocket cost) was the real expenditure patients paid. A negative correlation existed between self-paid DDC and number of DDDs for gefitinib (*R*^2^ = 0.7826, *P* = 0.0007, Fig. 4D), erlotinib (*R*^2^ = 0.5509, *P* = 0.0140, Fig. 4E) and icotinib (*R*^2^ = 0.8389, *P* = 0.0005, Fig. 4F).

### Influence of insurance and price on drug consumption

**Fig. 5:**
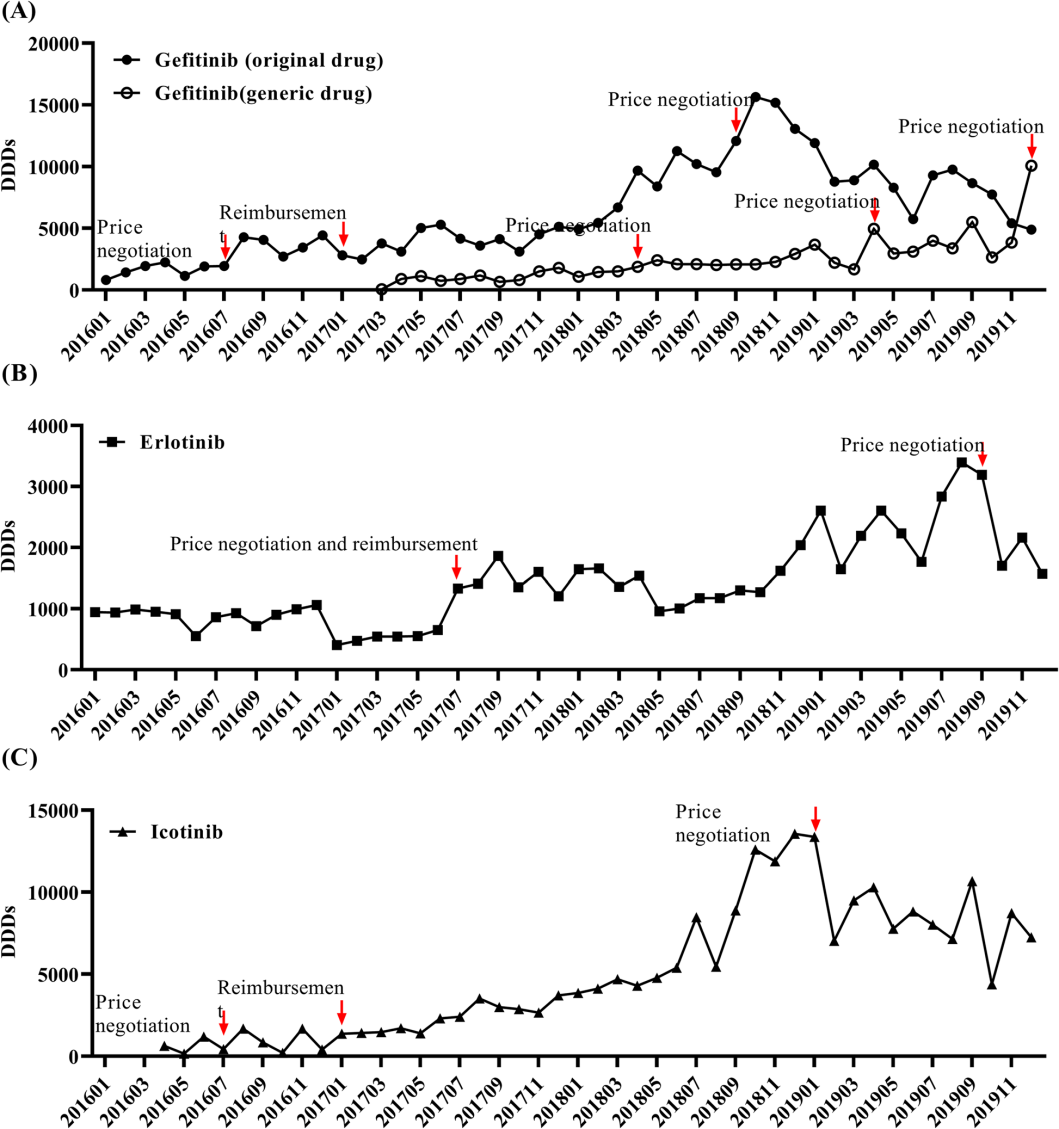
Monthly DDDs of Gefitinib, Erlotinib and Icotinib form January 2016 to December 2019. **A** Monthly DDDs of Gefitinib form January 2016 to December 2019; (**B**) Monthly DDDs of Erlotinib form January 2016 to December 2019; (**C**) Monthly DDDs of Icotinib form January 2016 to December 2019. DDDs, defined daily doses

Previous studies have reported that insurance reimbursement and drug price are impact factors of drug consumption. Hence, we analyzed their influence of reimbursement and price on the number of DDDs of TKIs. In July 2016, the DDC of gefitinib began to drop, and the number of DDDs kept increasing significantly in the subsequent 6 months (Fig. 5 and Table S1). Similar changes were also observed in icotinib. In January 2017, gefitinib and icotinib were covered by health insurance, with a reimbursement rate of 50%. Their number of DDDs both increased in the next 6 months (Fig. 5 and Table S1). In July 2017, erlotinib were covered by the national health insurance, and its DDC dropped. Thereafter, its number of DDDs increased by 175.55% (Fig. 5 and Table S1). In January 2019, afatinib and osimertinib were covered by the national health insurance, and their number of DDDs increased. In the same year, the prices of erlotinib and gefitinib fell, but their number of DDDs did not increase (Fig. 5 and Table S1).

## Discussion

Our study showed the obvious trends in the consumption and cost of EGFR TKIs in Nanjing from 2010 to 2019. Reimbursement, price negotiation, and generic drug replacement all played a role in increasing the number of DDDs. Our findings provide valuable evidence for the government and health organizations to adopt measures to improve drug availability and affordability.

EGFR TKIs have prolonged the survival of NSCLC patients with EGFR mutation [13]. However, the cost of TKIs has remained relatively high [13]. As reported in a US community-based study between January 1, 2008 and January 1, 2015, the total mean monthly cost during TKI therapy was $20,106 in advanced NSCLC [14]. In Serbia, the cost was 21.233€ in the first month after diagnosis among patients treated with TKI therapy, was much higher than the mean cost of all adenocarcinoma patients (1.317€) in 2013 [15]. In Southeast Asian low- and middle-income countries, only 15% of patients have access to erlotinib in 2011 [16]. In our study, the average monthly cost of EGFR TKIs was Ұ16,430.76, far beyond the average monthly household income [17]. This may explain the low and unchanged consumption of TKIs in 2010–2015.

DDC is an indicator that guides the price-making of pharmaceutical products on the market [11]. Previous studies have reported that price is a determinant of drug consumption [13, 18, 19]. Hence, we analyzed the correlation between DDC and the number of DDDs. As expected, the DDC was negatively correlated with the number of DDDs (Fig. 4). Fortunately, approaches have been taken to reduce drug cost, such as reimbursement policy, national price negotiation, generic drug replacement, low-price drug replacement.

More inclusive health insurance could significantly increase drug affordability. Previous studies have reported that Medicare Part D [20] and Medicaid [21] in America, public and private insurance in Canada [22] have increased drug consumption and decreased out-of-pocket costs. Similar results were also observed in our study. Gefitinib, icotinib and erlotinib were enrolled into the national health insurance in 2017 [5], and afatinib and osimertinib in 2019 [8]. Thereafter, the consumption of each TKI increased significantly (Fig. 5 and Table S1).

In response to increases in drug prices during the past few decades, Germany passed the Pharmaceutical Market Restructuring Act, known as AMNOG in 2011. In this act, price negotiations decreased treatment cost by an average of 24.5% [19]. In China, several rounds of price negotiations about EGFR TKIs have been held, which reduces DDC and increases the number of DDDs (Fig. 5 and Table S1).

Previous studies have shown that generic drug replacement could lead to significant cost reduction. In the study of Rwagitinywa J et al. [18], utilization of low-price generic antiretroviral drug significantly reduced the cost of HIV treatment in Denmark. But in France and Czech Republic, the drop in the cost of antiretroviral drugs is more related to the lower price of brand drugs than to the availability of generic drugs. In our study, the available generic gefitinib and China-made icotinib significantly increased the consumption of EGFR TKIs (Fig. 5 and Table S1).

There are some limitations in our study. First, the data contained no information on patients’ compliance with therapy; the term “consumption” is used for the quantity of drug prescribed but does not indicate how much the drug is administered. Second, we did not access the prescription switch between EGFR TKIs after reimbursement and price negotiation. Third, we did not access the clinical benefit of cost reduction.

## Conclusion

The consumption of EGFR TKIs was relatively low because of their high cost from 2011 to 2015 in Nanjing. The accessibility and affordability of these drugs have been improved by national policies since 2016, such as reimbursement, price negotiation and generic drug replacement. Accordingly, the DDC of EGFR TKIs decreased gradually, and the number of DDDs of EGFR TKIs increased annually ever since 2016. Further efforts are needed to translate the higher consumption of EGFT TKIs into clinical benefits.

## Supplementary Information

**Additional file 1:** **Table S1.** The influence of insurance and price on drug consumption.

## Data Availability

The datasets used and/or analyzed during the current study available from the corresponding author on reasonable request.
