# Supplementary material for: Consumption and cost trends of EGFR TKIs: influences of reimbursement and national price negotiation
Source: BMC Health Serv Res. 2022 Apr 1;22:431. doi: 10.1186/s12913-022-07868-9 (PMC8973903; doi:10.1186/s12913-022-07868-9)
Supplement: Supplementary file 1 — Additional file 1: Table S1. The influence of insurance and price on drug consumption. [file 12913_2022_7868_MOESM1_ESM.docx]

**Table S1 The influence of insurance and price on drug consumption**

| Drug | Change | Time | Average DDDsof 6 months before change (DDDs per month) | Average DDDs of 6 months after change (DDDs per month) | DDDs change (%) |
| --- | --- | --- | --- | --- | --- |
| Gefitinib (original drug) | DDC decreased from ¥ 510.00 to ¥ 235.80 | Jul 2016 | 1578.33 | 3471.67 | 119.96 |
| Gefitinib  (both) | Covered by medical insurance | Jan 2017 | 3471.67 | 4451.67 | 28.23 |
| Gefitinib (original drug) | DDC decreased from ¥ 235.80 to ¥ 228.00 | Sep 2018 | 9291.67 | 12770.00 | 37.43 |
| Gefitinib (generic drug) | DDC decreased from ¥ 176.00 to ¥ 158.40 | Apr 2018 | 1358.33 | 2095.00 | 54.23 |
| Gefitinib (generic drug) | DDC decreased from ¥ 158.40 to ¥ 80.00 | Apr 2019 | 2473.33 | 3985.00 | 61.12 |
| Gefitinib (generic drug) | DDC decreased from ¥ 80.00 to ¥ 27.50 | Dec 2019 | 3746.67 | 10080.00 | 169.04 |
| Erlotinib | Covered by medical insurance; DDC decreased from ¥ 601.24 to ¥ 195.00 | Jul 2017 | 529.67 | 1459.50 | 175.55 |
| Erlotinib | DDC decreased from ¥ 182.25 to ¥ 81.00 | Sep 2019 | 2503.67 | 2156.00 | -13.89 |
| Icotinib | DDC decreased from ¥ 396.43 to ¥ 199.86 | Jul 2016 | 658.00 | 874.00 | 32.83 |
| Icotinib | Covered by medical insurance | Jan 2017 | 873.83 | 1607.67 | 83.98 |
| Icotinib | DDC decreased from ¥ 199.86 to ¥ 192.15 | Jan 2019 | 10136.00 | 9461.67 | -6.65 |
| Afatinib | Covered by medical insurance | Jan 2019 | 129.79 | 1389.50 | 970.58 |
| Osimertinib | Covered by medical insurance | Jan 2019 | - | 5755.00 | - |
